# When Johnny Comes Marching Home

**Published:** 1918-11

**Authors:** 


### When Johnny Comes Marching Home Again.

The problem of the volunteer army surgeon in resuming private practice at the close of the war, has wisely been taken up in advance, both by the Surgeon General's office, the State and various other medical societies. The general experience of the past has been that it requires about four years to build up a living practice except when competition is practically nil on account of the small size of a village where also, acquaintance by and with or, at least knowledge of the existence of a physician, is easily acquired. Experience has also shown that it makes little difference whether the physician is a recent graduate, one of long practice but without acquaintance or even if he resumes residence where he has previously been known or has practiced, after a long interval. On the other hand, a brief absence, up to six months or a year does not have a serious destructive influence on practice, especially if for professional duty or for a good reason of any kind. For this reason, quite a number of medical men have urged that volunteer service be on a six months' basis but the argument against this plan, from the standpoint of the army or navy, is obvious.

The various plans for turning over private practice intact, in trust to a substitute, during the period of service, depend for success upon the actual survival of the old conception of a permanently loyal personal clientele. Without having attempted to gather statistics, we are somewhat sceptic as to the fundamental basis of this plan. The permanency of a practice differs somewhat according to longitude, in favor of the east; somewhat according to population, in favor of the smaller towns; considerably according to length of practice, in favor of the older men; obviously, largely according to nature of practice, whether general or special, surgical or medical, family or consulting, etc. In general, we are inclined to think that very few practices of men likely to serve with the army or navy for the war, can be handled as units, even if we assume good faith and altruism on the part of all concerned.

From the experience of the Civil War and with due regard to modern conditions, we believe that the young man who has not built up a considerable practice will make no sacrifice in volunteering, limiting the word sacrifice to purely professional considerations. He will receive, all things considered, as much as he would get in private practice, will gain a wide experience much of it available in subsequent civil practice, will be well disciplined and will gain a prestige which was of indubitable value after the Civil War. The surgeon, depending mainly on consultation and reference practice will sacrifice only what can be immediately reckoned by subtraction of salary from fees and will gain both in actual skill and in prestige, for the future. Some hold the opinion that the sacrifice is greater for older than for younger men, whatever the nature of their practice. From one standpoint this is true, as they will have less time in which to reap ultimate benefits. On the other hand, they are more likely to be prepared for final retirement, diversity of experience in life counts more with them than actual years, they or their heirs are more likely to receive the somewhat sinister benefit of various forms of pension.

What we want to emphasize particularly is that it is more important to exercise the general principles of fair play and consideration than to devise a theoretically perfect plan of caring for practices. For example, Dr. A, leaving for military service, turns over patient X, a unit in a suppositious permanent clientele, to Dr. B, using a special blank for that purpose. It is easily seen that X may prefer to go to Dr. C and so on down through several alphabets and, it is clear that Dr. C, having been passed over by Dr. A, may feel no obligation to him. Even Dr. B may in many instances claim in good faith that Z has already been his own patient or, at least, that he belongs to A no more than to D, G, M and N. The actual fact seems to be that a great many patients have no definite allegiance to any one physician and that in many families nearly every member has his or her own physician, more or less subject to change. So far as most special and consulting practices are concerned, no scheme of turning over patients can be devised that will work without friction.

The real solution of the problem, it seems to us, is not to attempt to devise any formal system of substitute attendance but to cultivate a spirit of fair play, generosity and appreciation of sacrifice, that will give Dr. A, on his return to private practice, not only a cordial welcome but practical assistance in resuming his work. This can be done not merely by individual displays of good will within the profession but by whole-hearted and sincere appeals from the profession as a body to the laity. On the other hand, there are practical limits to such expressions of patriotic appreciation. The man who remains at home and attends to the increased medical work, pays increased taxes and contributes to various philanthropies of war, is by no means necessarily a slacker, he should not be required to do an excessive amount of work without pay, nor should he be made a post bellum sacrifice. It is probable that the war, even with no exaggerated ideas of the direct killing off of physicians, will in various ways, tend to bring about an equilibrium of demand and supply as regards the medical profession. We shall probably maintain a good sized military establishment for some years and this, in turn, will require a permanent increase in the army medical corps. Various undertakings, requiring medical services, on a contract basis under the control of the government, will undoubtedly be initiated or greatly increased as war measures and will continue after the war. There are many ways in which one or another branch of the government can provide for the more or less permanent employment of the surgeons whom we regard in anticipation as to be thrown upon their own resources at the conclusion of hostilities. A reasonable effort by the national and local governments to provide places for surgeons on the conclusion of their service, combined with popular and professional good will, expressed in practical ways, ought to solve the problem. And, finally, we must not forget that war demands sacrifices, which can not be distributed with absolute equality nor nullified by systems of insurance against sacrifice.

